# micropan: an R-package for microbial pan-genomics

**DOI:** 10.1186/s12859-015-0517-0

**Published:** 2015-03-12

**Authors:** Lars Snipen, Kristian Hovde Liland

**Affiliations:** 1 Department of Chemistry, Biotechnology and Food Sciences, Norwegian University of Life Sciences, P.O. Box 5003, N-1432 Ås, Norway; 2 Nofima - Norwegian Institute of Food, Fisheries and Aquaculture Research, Osloveien 1, N-1430 Ås, Norway

**Keywords:** Pan-genome, R, Clustering, Prokaryotes

## Abstract

**Background:**

A pan-genome is defined as the set of all unique gene families found in one or more strains of a prokaryotic species. Due to the extensive within-species diversity in the microbial world, the pan-genome is often many times larger than a single genome. Studies of pan-genomes have become popular due to the easy access to whole-genome sequence data for prokaryotes. A pan-genome study reveals species diversity and gene families that may be of special interest, e.g because of their role in bacterial survival or their ability to discriminate strains.

**Results:**

We present an R package for the study of prokaryotic pan-genomes. The R computing environment harbors endless possibilities with respect to statistical analyses and graphics. External free software is used for the heavy computations involved, and the R package provides functions for building a computational pipeline.

**Conclusions:**

We demonstrate parts of the package on a data set for the gram positive bacterium *Enterococcus faecalis*. The package is free to download and install from The Comprehensive R Archive Network.

**Electronic supplementary material:**

The online version of this article (doi:10.1186/s12859-015-0517-0) contains supplementary material, which is available to authorized users.

## Background

As a result of accelerated development of sequencing technologies over the last decades, prokaryotic genomes can now be sequenced in short time and at small cost. This has made it popular to sequence many strains within the same species, to investigate the diversity, to look for a core set of genes common to all strains, to be able to distinguish pathogenic from non-pathogenic genotypes, etc. [1-5].

It has long since been recognized that the diversity between prokaryotic strains is much greater than between, say, individuals in a human subpopulation. All humans share, more or less, the same genes, and the differences between individuals are often found in the regulatory parts of the genome. In contrast, two different strains of *E. coli* may be many times more diverse than humans and chimpanzees, with perhaps 20% of the genes in one strain lacking in the other (e.g. [6,7]). For this reason, the set of unique genes found in the entire *E. coli* population is many times larger than what we observe in a single genome, and this collection of all genes utilized by a species was by [8] denoted the ‘pan-genome’.

Microbial comparative genomics has in most cases focused on the core genes, i.e. housekeeping genes conserved across all strains [9]. In pan-genomics we consider all genes, also those referred to as accessory genes. In all cases, a ‘gene’ is actually a gene family or cluster of similar sequences, and we only consider the protein coding genes. A gene family is usually synonymous to a collection of orthologs, i.e. genes playing identical roles in different genomes. Since the grouping we come up with in pan-genomics does not always correspond to the classical gene family, we prefer the term *gene cluster* instead of gene family. Despite the central role of such clusters or families, there has never really been any consensus on how to compute them [10], and in the tool set presented here we have implemented several options.

In this paper we describe and illustrate some functions and opportunities of the micropan R-package [11] by using public data for the species *Enterococcus faecalis*. *E. faecalis* is a multifaceted Gram-positive bacterium with an intimate relationship to human health and disease [12]. This species is among the first to colonize the gastro intestinal tract of newborns [13], and for most people *E. faecalis* constitutes a commensal of the normal intestinal microflora [14]. Remarkably, while certain strains are used as probiotics [15], *E. faecalis* is also a prominent cause of nosocomial infections [16]. Hence, this extreme diversity in phenotype among different strains motivates an investigation of the species’ pan-genome.

## Implementation

Within the package there is a document (casestudy. pdf) giving a detailed description, with R-code, of a case study example that can serve as a template for most studies. Here we give an overview of the package implementation.

We have chosen to use R as the ‘workbench’ for making microbial pan-genome analyses. R is a free and platform-independent tool much used in bioinformatics, and it requires only very modest programming skills. Inside R there is a huge array of other packages and solutions available, making the possible combinations endless. A long list of basic and complex statistical analyses can be made, as well as graphical displays of all kinds. The micropan package offers a set of functions specifically designed for microbial pan-genomics, but it is quite natural to also make use of other facilities in R once the basic computations are made.

In addition to R, we also make use of some external software, typically for heavy computations. All external software used by the micropan package are free and platform-independent, and the package vignette gives an overview of how to obtain them. Once installed, the external software are operated by using micropan R-functions. This means you never leave the R-environment while doing the analyses, but still make use of these extremely efficient computing engines.

### The pan-matrix

A central data structure in a pan-genome analysis is the *pan-matrix*. Any pan-genome analysis is typically divided into two phases; first, the heavy computations required to establish the pan-matrix, and then the more interesting analyses where the pan-matrix is often used as the input.

Any pan-genome study requires a set of FASTA-files containing the protein sequences of all genes in each genome. The micropan package has functions for downloading genomes (complete or draft) from NCBI [17], using the Entrez programming utilities [18], and if gene calling is needed the function prodigalPredict can be used to call upon the Prodigal gene finder [19] as an external software.

To compute the pan-matrix, a large number of protein sequence comparisons must be made, which is the computational bottleneck of a pan-genome study. We have chosen the software BLAST+ or HMMER3 [20-22] for this job, highly optimized standard tools in the bioinformatics toolbox. These are invoked from R by the functions blastAllAll and hmmerScan in the micropan package. The sequence comparisons are still time-consuming, but they are only done once for each data set since all results are stored on files in a specified output-folder. If data sets are extended, only computations involving the new genomes are needed. On a multi-core computer you can easily increase the computational speed by running multiple R-sessions in parallel. We have designed the functions not to overwrite existing result files, allowing multiple R-sessions to write to the same output-folder.

Based on the result files from the heavy computations, sequences are clustered into gene clusters. The micropan package contains functions for doing this in various ways. Clustering genes into classical gene families (clusters of orthologs) is a common approach [23], and means all sequences must be compared to all sequences in a *direct* way (pairwise alignments). This is an option in the micropan package as well, based on results from blastAllAll. Distances between sequence *i* and *j* are computed as

(1) $$ D(i,j) = \frac{1}{2}\left[ 2-S(i;j)/S(j;j)-S(j;i)/S(i;i)\right]  $$

where *S*(*i*;*j*) is the BLAST score for the alignment between the sequences when *i* is used as query and *j* as database sequence, and vice versa for *S*(*j*;*i*). The self-scores *S*(*j*;*j*) and *S*(*i*;*i*) are used to get the relative score (between 0 and 1), and the distance is simply the average lack of relative score. Gene families are found by a hierarchical clustering where linkage functions and cutoff thresholds can be specified.

However, any all-versus-all type of comparison suffers from the quadratic scaling, i.e. the computational load is *O*(*G* ^2^) where *G* is the number of genomes involved. It is feasible for a smallish number of genomes, but considering the ever-growing number of sequenced strains, it has some daunting perspectives. Already the approach would prove difficult for *Escherichia coli*. At the time of writing there are 2267 strains available at NCBI. Producing the more than 5 million (2267^2^) BLAST result files would take weeks given ordinary computing resources, and in the meantime the number of *E. coli* strains has increased further!

An alternative to clustering based on orthologs is to scan all genes for protein domains using HMMER3, as suggested by [24], and then cluster them by their ordered sequence of non-overlapping domains. The whole idea of this *indirect* comparison is to first compare all proteins to some reference set of sequences, e.g. a database of protein domains, and then cluster them based on how they look in this sequence subspace. This approach scales linearly in the number of genomes, and is more robust to gene prediction errors since an accurate start-codon prediction is not as critical as for the direct comparison approach.

Finally, the pan-matrix is constructed from the clustering results based on either BLAST+ or HMMER3 comparisons. The pan-matrix has one row for each genome and one column for each gene cluster, and the value in cell (*i*,*j*) is the number of copies of gene cluster *j* found in genome *i*.

### Exploring pan-genomes

Pan-genome size can be estimated by methods previously suggested [25-27]. The Chao lower bound estimate of pan-genome size is a conservative estimate, i.e. it tends to be on the smaller side of the true size. Fitting a binomial mixture model will also produce a conservative estimate of pan-genome size, as well as an estimate of the core size. Such models also give a nice graphical view of the composition of the pan-genome with respect to gene types, ranging from core genes (always or almost always present), shell genes (often present, but lacking in subsets of genomes) to cloud genes (observed in only a few genomes).

Pan-genome openness/closedness can be estimated as suggested by [28], using a Heaps law type of model. This model is fitted to rarefaction curves for the data, and the display of such curves is also implemented.

Another quantity describing pan-genome diversity is the genomic fluidity suggested by [29], also implemented as a function in the micropan package. The fluidity is very similar to a Jaccard distance, which is a measure of overlap between genomes with respect to their gene clusters. Both Jaccard distances as well as Manhattan distances between genomes can be computed. The functions distManhattan and distJaccard will by default consider only presence and absence of any gene cluster in a genome, i.e. transform the pan-matrix values to 0^′^ *s* or 1^′^ *s*.

**Figure 1:**
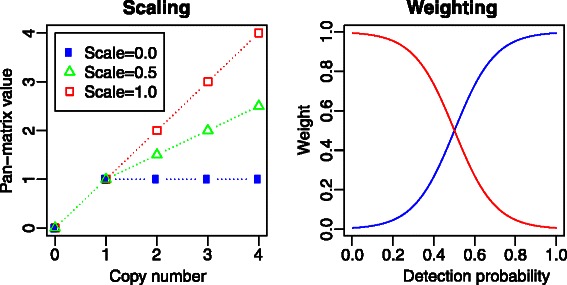
**Scaling and weighting.** The left panel shows how the scaling parameter affects the values of the pan-matrix when computing distances between genomes. With no scaling (default) copy numbers beyond 1 are ignored, i.e. for a gene cluster occurring in 1 or more copies the pan-matrix will only have the value 1. If the scale is 1 (maximum) the pan-matrix value is identical to the copy number. Any scale value between 0 and 1 will produce intermediate pan-matrix values. The right panel shows two gene cluster weight strategies available in the geneWeights function. The blue curve is the *shell* weighting, giving large weight to gene clusters occurring frequently (large detection probability). The red curve is the *cloud* weighting, emphasizing rarely occurring gene clusters.

The distManhattan function may also consider copy number variation. The argument scale is used for this, as demonstrated in Figure 1. By default scale =0.0 and a gene cluster is only counted as present or absent regardless of the actual number of copies for that cluster. Using scale =1.0 means all copy number differences are equally important. Any value between 0.0 and 1.0 will put some emphasis on copy number changes, but going from 0 to 1 copy is still the most severe difference. It is also possible to give weights to certain gene clusters. By default all gene clusters have weight 1.0, but by lowering this certain gene clusters can be partially or totally ignored in the distance computations. Figure 1 also illustrates some built-in weighting options.

Based on some function for computing distances (mentioned above, or made by the user) the genomes can be displayed in pan-genome trees as described in [30]. An alternative display is by principal component analysis (PCA). A pan-matrix, like any matrix, can be used as input in a PCA, and by plotting the genomes in a truncated score-space we may get an impression of how the genomes are located relative to each other. A PCA biplot may also reveal which gene clusters are the best discriminators between (groups of) genomes. A PCA on a pan-matrix will most typically indicate if genomes tend to cluster. The panpca function can also take arguments for copy number scaling and gene cluster weighting, as mentioned for distManhattan above.

## Results and discussion

To demonstrate some facets of the micropan package we have used publicly available data for the species *E. faecalis*. We have chosen this rather large data set since it is typical of what we will face in near future for all species. Within the micropan package a case study document demonstrates the approach on a very small data set, to make all computations fast for a do-it-yourself exercise.

First, genome data for 342 *E. faecalis* genome sequencing projects were downloaded from NCBI by entrezDownload. Only 5 of these genomes were complete and the rest were draft genomes. Next, we called protein-coding genes in all genomes using the prodigalPredict function. In the 342 FASTA-files of amino acid sequences the median number of proteins was 2916 with a range of 2428 to 3482, and close to 1 million sequences in total.

In the present study, since we have as many as 342 genomes, we decided to compare sequences by their protein domain sequences. All proteins were scanned against the Pfam-A database [31] using the hmmerScan function. A total of 2318 unique Pfam-A entries produced hits, with around 3500 hits per genome. On average 84*%* of the proteins in a genome contained some Pfam-A domain(s). Each sequence having a hit in Pfam-A was clustered together with all other sequences having the exact same domains in the exact same non-overlapping order, using the dClust function. Clearly, a drawback of this approach is that the 16*%* proteins without hits in the Pfam-A database are excluded from the analysis. Using only domain sequences we maintain a very high degree of specificity (including only real genes in the analysis) at the cost of a lower sensitivity. This we find fruitful in pan-genome studies, where the inclusion of some ‘rubbish genes’ in every genome would assemble into a large pool of (seemingly) ORFan genes in the entire pan-genome. The sensitivity can be improved to some degree by extending the domain database, e.g. including data from CDD and/or InterPro [32,33].

**Figure 2:**
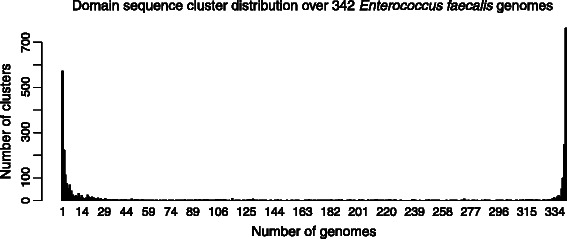
**Presence/absence distribution of clusters.** The bar chart shows the number of domain sequence clusters found present in 1,2,…,342 of the *E. faecalis* genomes. This is the default chart when plotting a pan-matrix in the micropan package.

The pan-matrix was constructed from the domain sequence clusters. We found 3111 unique domain sequence clusters in the entire data set, with an average of 1465 in a single genome. Domain sequence clusters are larger and more coarse groups than classical gene families, reflected in the fact that there are approximately half as many clusters as there are protein sequences in an average *E. faecalis* genome. Some clusters are huge; the largest is characterized by the single domain PF00005.22, which is an ABC Transporter type of protein. All *E. faecalis* genomes contain many of these genes. Figure 2 shows the micropan default graphical display of the pan-matrix, a barplot listing the number of clusters found in 1,2,…,342 genomes. We found 762 clusters present in all 342 genomes (rightmost bar) and at the other end there are 572 clusters unique to one genome only. Thus, on average every genome has around 2 domain sequence clusters never seen in any other *E. faecalis* genome.

**Figure 3:**
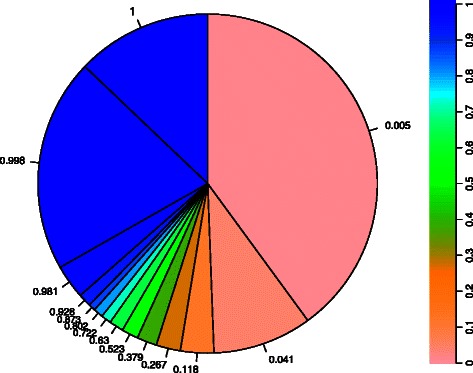
**Visualization of the pan-genome.** The pie symbolizes the entire pan-genome, and the 14 sectors correspond to various groups of gene clusters, where the color of the sector shows the detection probability, also indicated by the numbers. The blue sectors are highly conserved (core) genes. These are found in (almost) all genomes. The greenish sectors are accessory (shell) genes found in (large) subsets of genomes. The orange/pink sectors are the rarely occurring (cloud) genes. Most of these have not yet been described. Only one or a few such genes are found in each genome, but if we keep on sequencing they will make up a large proportion of the pan-genome.

Based on the pan-matrix we fitted a binomial mixture model using the binomixEstimate function [27] describing the *E. faecalis* pan-genome. This function estimates a series of mixture models of increasing complexity, and uses the Bayesian Information Criterion (BIC, [34]) to find the optimum. For this data set the optimum was found at 14 components, i.e. gene clusters should be divided into 14 groups with respect to how frequently they appear in the genomes. The pie-chart visualization of the fitted binomial mixture model is perhaps the most descriptive visual summary of the pan-genome. Gene clusters are assembled into groups having specific detection probabilities, and the relative distribution of these indicate how the species’ gene content looks like. In Figure 3 we show this picture of the *E. faecalis* pan-genome.

The core gene clusters corresponds to the darkest blue sector, with detection probability 1. There are also two other sectors with almost the same dark blue, and detection probabilities close to 1. In practice, we would say these are also core genes. They lack in some of the 342 genomes, but since these data contain mostly incomplete draft genomes we do not expect 100% detection even for real core gene clusters. Thus, the core genes make up roughly one third of the *E. faecalis* pan-genome. These gene clusters comprise the stable part of the pan-genome. At the other end we notice the large sector of ‘cloud’ gene clusters (pink). If we also include the sector with detection probability 0.041 we see that such gene clusters make up half the pan-genome. Even if they are many, they are rarely found in a genome (low detection probability). The history and function of such genes is interesting from a biological viewpoint, but is of less importance for describing the population of *E. faeclis* strains. From Figure 3 we also note that around 10-15% of the pan-genome are ‘shell’ gene clusters (greenish colors), being present in many, but not all, genomes. These gene clusters are interesting. In this segment we find the really strong signals with respect to which strains are more or less similar. If we should design a typing scheme for this species, we would certainly consider these gene clusters as important.

Estimates of the full pan-genome size was obtained from the fitted mixture model as well as the chao [25] function in the micropan package. Both produce conservative estimates of how many clusters we should expect to see if we continue to sequence more strains, the results were 3327 and 3845, respectively. Both these methods assume the pan-genome is closed, i.e. its size has an upper bound. The heaps function was used to indicate if this is actually the case. The Heaps law model parameter *α* was estimated to 0.82, indicating an open pan-genome, the threshold being 1.0, see [28] for details. We will, however, not conclude from this that the *E. faecalis* pan-genome is ‘infinite’. An ‘open’ pan-genome must be interpreted as a size much bigger than what we have seen so far. The pan-genome size estimation is by nature a very difficult problem since it implies an extrapolation way beyond the present data. This must always be acknowledged and warrants a caution on making statements about pan-genome open/closedness.

The estimation of a pan-genome size is a variant of the ‘number of unseen species’ problem that dates back at least to [35]. The methods implemented in the current version of this R package are just some out of several possible [36]. Recent investigations have demonstrated that both these and some other methods have their flaws [37]. More methodological research should be devoted to this problem, and future versions of this R package will be updated accordingly. Specifically, methods incorporating information about genome relatedness should have a potential for improving such estimates. This has recently been tried out by [37,38], and similar ideas from related problems should also be explored [39].

Given the pan-matrix, we can construct a pan-genome tree, as described in [30]. Such trees illustrate the difference in gene cluster content between genomes. For 342 genomes any such tree requires too much space for being displayed in this paper, but in Additional file 1: Figure S1 we have included such a (unweighted and unscaled) tree for all genomes.

**Figure 4:**
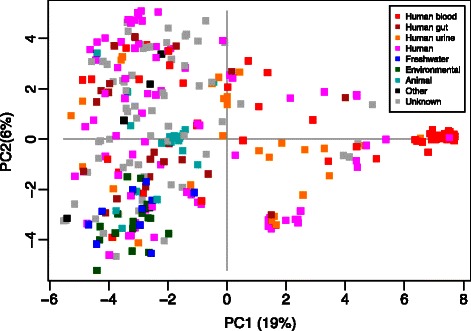
**PCA on a pan-matrix.** Each square marks a genome in the two first principal components (directions) of the pan-matrix space. The colors indicate the type of environment from which the genome has been sampled. Note that the magenta genomes are isolated from humans, but without specifying blood, gut or urine. The percentages in the axis labels show how much of the total pan-matrix variation is seen along each principal component.

An alternative display of the genomes is obtained by a principal component analysis (PCA). A pan-matrix can be used in a PCA to rotate and truncate its column-space, and plotting the genomes in the first 2-3 dimensions will reveal the dominant differences between them. In Figure 4 we have used the panpca function together with plotScores to show how the genomes are located in the space spanned by the two first principal components. We have used the default scale =0 and no weighting. The genomes are colored according to the environment they have been isolated from, collected from the BioSample database at NCBI [40]. Only 25% of the total variation is seen along these two directions, but there is still some interesting information to be found. We notice that the first principal (horizontal) axis is spanned by the tight cluster of strains from human blood on the right. This group is dominated by a subset of strains from the Enterococcus Illumina PacBio initiative, Broad Institute (broadinstitute.org). This project has provided many of the genomes shown here, and only a subset of them forms this very distinct cluster. There are other tendencies of clustering as well, especially a subset of human (urine) strains around position (2,−3) in the coordinate system. We also notice that all environmental and freshwater isolates (green and dark blue) are located in the lower left quadrant. This indicates that the present/absent patterns of domain sequence clusters is far from random and clearly has a typing potential.

**Figure 5:**
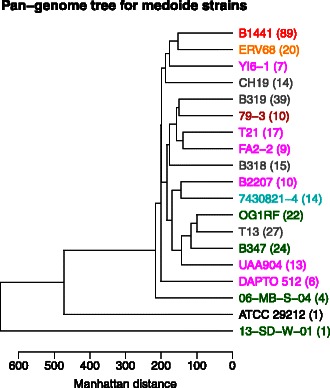
**Pan-genome tree for medoide genomes.** This pan-genome tree was constructed using unscaled and unweighted Manhattan distances between the medoide genomes after partitioning all genomes into 19 groups (see the text for details). A medoide is the ‘central’ genome in the group. The number of genomes in each group is shown in the parenthesis behind each name. The colors indicate the dominating environment in each group, see Figure 4 for color-code. The gray are mixes without any dominating environment.

Since Figure 4 indicates some clustering, we decided to partition the genomes and display the central genome from each group in a pan-genome tree. We first used the distManhattan function to produce a distance table. To illustrate the use of other R-facilities, we used the cluster package for the partitioning. We used the Partitioning Around Medoids method [41], clustering the genomes into 2,3,…,100 clusters. For each number of clusters we computed the silhouette values [41]. The silhouettes measure how well a genome belongs to its assigned cluster compared to its nearest neighbor cluster. We computed the mean of the 25% smallest silhouettes in each case, and found that this reaches its maximum at 19 clusters, as shown in Additional file 2: Figure S2. Thus, the data suggest that the 342 genomes can be separated into 19 groups. From each group we extracted the medoide genome and constructed a pan-genome tree, using the panTree function and displayed it in Figure 5. Each group is represented by the medoide genome name, while the color represents the dominating environment of the genomes in the group. We notice there is one big group of human blood genomes (red, B1441 as medoide), one with human urine (orange, ERV68) and one with human gut (brown, 79-3). There are 6 more groups of human isolates (magenta). The three uppermost clades in the tree (red, orange and magenta) contain only human isolates. One cluster is dominated by animals (light blue, 7430821-4) and 4 groups are environmental/freshwater (green). The gray groups are mixes without any dominating environment. Two strains, the environmental isolate 13-SD-W-01 and the lab-strain ATCC 29212, are extremely different from all others. This partitioning procedure has some resemblances to the definition of clonal complexes based on MLST-analyses [42], but here we use all gene clusters instead of a small subset of core genes. Supervised learning methods could now easily be implemented to design whole-genome based typing procedures for *E. faecalis*, but this is beyond the scope of this application note.

## Conclusions

We have presented an R package including a number of functions for exploring microbial pan-genomes. The implementation uses R as the working environment, but exports heavy computations to external software. Within the package is a small case study example for the user to explore. In this paper we have used a larger data set for *Enterococcus faecalis* to demonstrate some of the facets in this package. We have also used other functions and packages available in R on the *E. faecalis* data to highlight these possibilities from within the R computing environment.

## Availability and requirements

The R package is available for free from The Comprehensive R Archive Network (CRAN, [43]). It is most easily obtained by starting R and runninginstall.packages(~micropan~, repos=~http://cran.r-project.org/~) in the console window.**Project name:** micropan **Project home page:** Search for micropan on CRAN**Operating systems:** Platform independent**Programming language:** R**Other requirements:** For full functionality the system must also have available the BLAST+ software [20], the HMMER3 software [21,22] and the Prodigal software [19]. **License:** GPL-2

## Additional files

Additional file 1
**Figure S1 - Pan-genome tree for all genomes.** An unscaled and unweighted pan-genome tree for all the 342 *E. faecalis* genomes used in the example study. The colors of the leaves reflect the environment from which the genome has been sampled, and the color code is shown in Figure 4.

Additional file 2
**Figure S2 - silhouette values.** Genomes were clustered into 2,3,…,100 groups using the Partitioning Around Medoids (PAM) method in the cluster package in R. For each number of clusters the mean of the 25% smallest silhouette values are shown. The maximum value is reached at 19 clusters, indicating the best possible grouping according to this criterion.
