# Supplementary figures and images for: micropan: an R-package for microbial pan-genomics
Source: BMC Bioinformatics. 2015 Mar 12;16:79. doi: 10.1186/s12859-015-0517-0 (PMC4375852; doi:10.1186/s12859-015-0517-0)

Enterococcus faecalis pan-genome tree

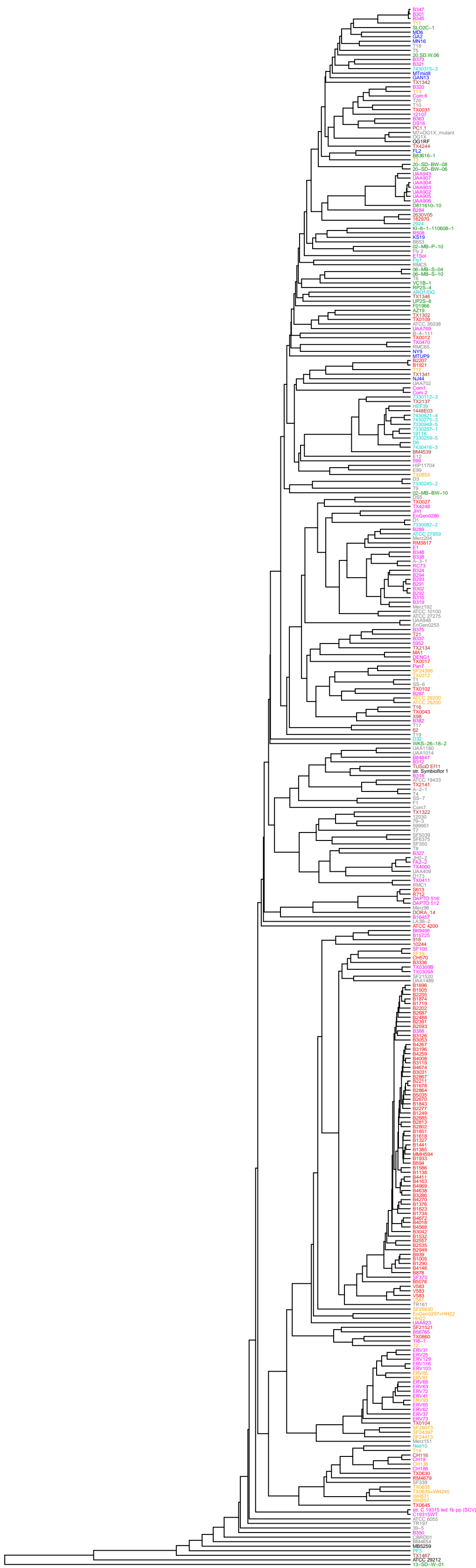

Manhattan distance

Supplement: Additional file 1 — Figure S1 - Pan-genome tree for all genomes. An unscaled and unweighted pan-genome tree for all the 342 E. faecalis genomes used in the example study. The colors of the leaves reflect the environment from which the genome has been sampled, and the color code is shown in Figure 4. [file 12859_2015_517_MOESM1_ESM.pdf]

Silhouette value

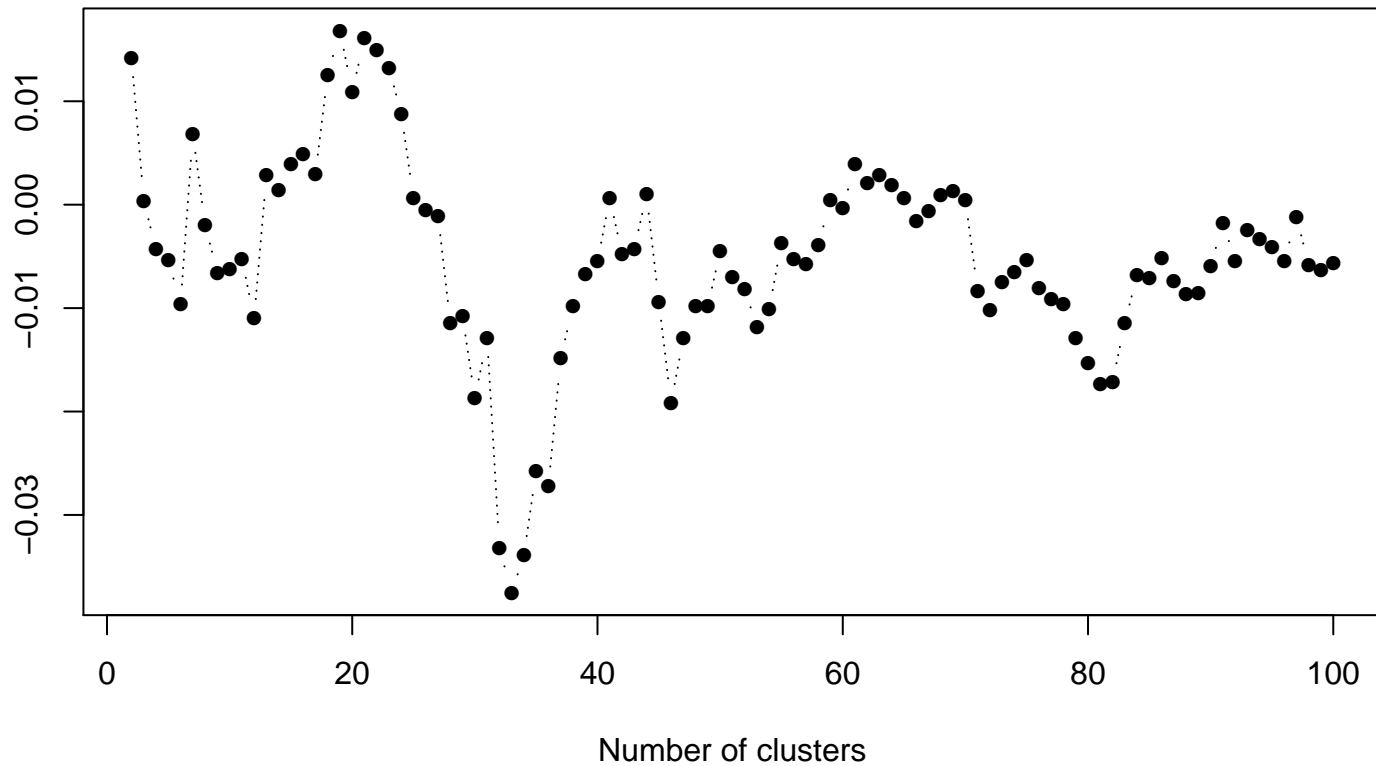

Supplement: Additional file 2 — Figure S2 - silhouette values. Genomes were clustered into 2,3,…,100 groups using the Partitioning Around Medoids (PAM) method in the cluster package in R. For each number of clusters the mean of the 25% smallest silhouette values are shown. The maximum value is reached at 19 clusters, indicating the best possible grouping according to this criterion. [file 12859_2015_517_MOESM2_ESM.pdf]
